# Energy Metabolism in the Failing Right Ventricle: Limitations of Oxygen Delivery and the Creatine Kinase System

**DOI:** 10.3390/ijms20081805

**Published:** 2019-04-12

**Authors:** Ewan D. Fowler, David Hauton, John Boyle, Stuart Egginton, Derek S. Steele, Ed White

**Affiliations:** 1 Multidisciplinary Cardiovascular Research Centre, University of Leeds, Leeds LS2 9JT, UK; david.hauton@chem.ox.ac.uk (D.H.); medjpb15@gmail.com (J.B.); s.egginton@leeds.ac.uk (S.E.); D.Steele@leeds.ac.uk (D.S.S.); E.White@leeds.ac.uk (E.W.); 2 Cardiac Research Laboratories, School of Physiology, Pharmacology and Neuroscience, University of Bristol, Bristol BS8 1TD, UK; 3 Metabolomics Research Group, Chemistry Research Laboratory, University of Oxford, Oxford OX1 3TA, UK

**Keywords:** myocardial hypoxia, creatine kinase, pulmonary artery hypertension, beta blocker, monocrotaline, right ventricle failure, heart failure

## Abstract

Pulmonary arterial hypertension (PAH) results in hypertrophic remodeling of the right ventricle (RV) to overcome increased pulmonary pressure. This increases the O_2_ consumption of the myocardium, and without a concomitant increase in energy generation, a mismatch with demand may occur. Eventually, RV function can no longer be sustained, and RV failure occurs. Beta-adrenergic blockers (BB) are thought to improve survival in left heart failure, in part by reducing energy expenditure and hypertrophy, however they are not currently a therapy for PAH. The monocrotaline (MCT) rat model of PAH was used to investigate the consequence of RV failure on myocardial oxygenation and mitochondrial function. A second group of MCT rats was treated daily with the beta-1 blocker metoprolol (MCT + BB). Histology confirmed reduced capillary density and increased capillary supply area without indications of capillary rarefaction in MCT rats. A computer model of O_2_ flux was applied to the experimentally recorded capillary locations and predicted a reduction in mean tissue P_O2_ in MCT rats. The fraction of hypoxic tissue (defined as P_O2_ < 0.5 mmHg) was reduced following beta-1 blocker (BB) treatment. The functionality of the creatine kinase (CK) energy shuttle was measured in permeabilized RV myocytes by sequential ADP titrations in the presence and absence of creatine. Creatine significantly decreased the K_mADP_ in cells from saline-injected control (CON) rats, but not MCT rats. The difference in K_mADP_ with or without creatine was not different in MCT + BB cells compared to CON or MCT cells. Improved myocardial energetics could contribute to improved survival of PAH with chronic BB treatment.

## 1. Introduction

Pulmonary arterial hypertension (PAH) arises when the vascular resistance in the normally low pressure, high compliance pulmonary circulation increases due to muscularization or occlusion of pulmonary arterioles [1,2]. The increased load on the right ventricle (RV) causes adaptive remodeling, such as hypertrophy of RV myocytes, increased heart rate, and increased reliance on inotropic beta-adrenergic signaling pathways [3]. These changes initially preserve cardiac output at the cost of increased heart rate and energy expenditure [4,5]. Chronic elevated beta-adrenergic signaling reduces beta-adrenoceptor expression, which compromises contractile reserve [6,7,8]. Eventually the RV is unable to meet demands and RV failure results [9].

It is believed that problems in energy supply could lead the heart to become “energy starved”, due to its continual high rate of ATP turnover [10]. In human heart failure, lower levels of high-energy phosphates like ATP and phosphocreatine (PCr) are associated with worse outcomes [11]. The causes of this are unknown; however, there is evidence of myocardial hypoxia in PAH, as well as left heart failure shown by increased expression of hypoxia-inducible factor-1 (HIF-1) and vascular endothelial growth factors (VEGFs) [12,13,14,15,16]. This may drive a shift in substrate utilization from fatty acid oxidation to glycolysis in both animal and human PAH [17,18].

Myocardial hypoxia in PAH could be explained by reduced right coronary artery flow in proportion to increased RV mass and increased heart rate, which decreases coronary perfusion time [5,19]. Increased myocyte diameter is also predicted to reduce tissue oxygenation, due to increased diffusion distance from capillaries [20]. In human PAH, capillary density was reduced in the failing RV with a reduction in pro-angiogenic miR-126 expression, whereas injection of miR-126 in a rodent model of PAH increased capillary density and cardiac output [21]. Depending on the stage of disease, there may also be increased expression of VEGF inhibitors and decreased expression of angiopoetin-1 and apelin proteins involved in capillary maintenance [22]. Hypoxia will reduce mitochondrial ATP synthesis and limit the cell’s ability to perform work; therefore, reducing myocardial hypoxia could be a therapeutic target for PAH.

To work efficiently, ATPases like sarco/endoplasmic reticulum Ca^2+^ ATPase (SERCA) and myosin ATPase require a high free energy of ATP hydrolysis, achieved by a large ratio of ATP (~5 mM) to ADP (<50 μM). This is maintained in the vicinity of ATPases by cytosolic creatine kinase (CK), which reversibly catalyzes the transfer of high-energy phosphate from phosphocreatine (PCr) to ADP, producing ATP and creatine (Cr). CK located in the mitochondrial intermembrane space (CK-mt) generates PCr and promotes ATP synthesis by maintaining a high mitochondrial [ADP] [23,24]. This shuttling of energy from mitochondria to cellular compartments using the CK system becomes particularly important for cell survival during hypoxia and with moderately increased O_2_ flux [25]. However, expression of CK isoforms is reduced in PAH and left heart failure, which is associated with impaired myofilament function and reduced coupling to respiration [26,27,28,29]. This raises the prospect that improving CK function may benefit a failing RV, and therapies which do so should be investigated.

Beta-adrenergic receptor blockers (BB) are not currently recommended as a treatment for PAH; however, recent clinical [30,31,32,33] and preclinical evidence suggests they may be safe or beneficial [34,35,36,37,38]. We recently established a BB treatment regime in a monocrotaline (MCT) rat model of PAH, and it slowed the progression to heart failure [37]. Notably, BB ameliorated MCT RV myocyte hypertrophy and showed trends towards improved expression of CK isoforms. Our aim for this study was to investigate whether RV hypoxia and CK-mediated mitochondrial respiration were improved in MCT rats following chronic BB treatment.

## 2. Results

PAH was initiated by a single intraperitoneal injection of monocrotaline into male Wistar rats (200 ± 20 g). We employed a three-group study design, consistent with previous publications [34,35,36,37,38]. MCT-injected rats were treated daily with either the beta-1-selective blocker metoprolol (10 mg/kg; MCT+BB group) or with a vehicle (sucrose solution; MCT group) beginning on day 15 post-MCT injection [37]. Saline injected control rats (CON group) were treated with the vehicle. MCT rats were killed when showing symptoms of heart failure [37]. MCT + BB and CON rats were killed on the median survival day of MCT rats (23 ± 1 days) as time-matched treatment groups. Frozen tissue for histology and myocytes were isolated from the same cohort of animals used in [37], which also contains detailed in vivo characterization of the BB treatment model.

The heart weight to body weight ratio was greater in MCT rats than CON rats, mainly due to increased RV weight (Table 1). The heart and RV weight of MCT + BB rats was significantly less than MCT rats, consistent with a reduction in RV myocyte hypertrophy [37]. Lung weight was greater in MCT and MCT + BB rats than CON, possibly indicative of pulmonary edema arising from increased pulmonary artery systolic pressure.

**Figure 1:**
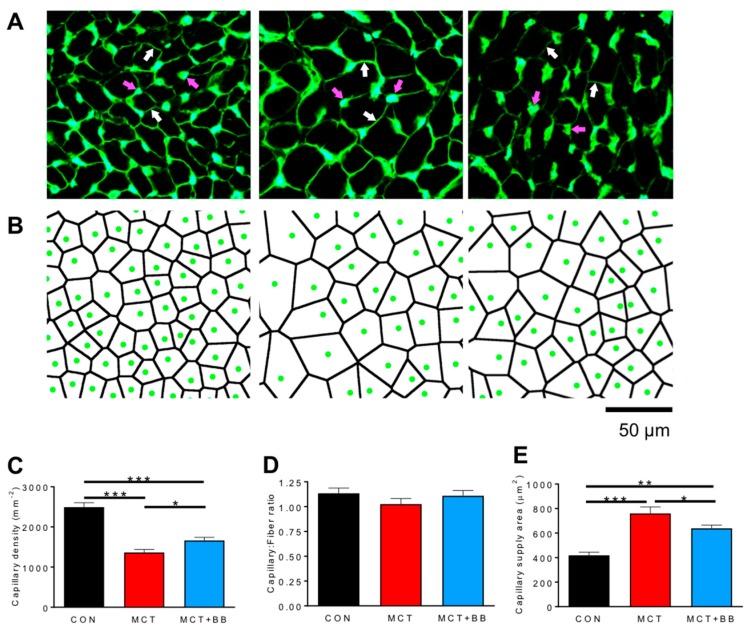
Capillary density is decreased and capillary supply area in the right ventricle (RV) is increased by MCT treatment. (**A**) Exemplar images showing intense fluorescent labelling of capillaries (magenta arrows) and less intense labelling of sarcolemma (white arrows) with FITC-conjugated lectin. (**B**) Capillary locations were digitized (green circles), and the area of tissue supplied by each capillary was calculated by Voronoi tessellation (black lines). (**C**) Capillary density was reduced in MCT and less so in MCT+BB compared to CON. (**D**) There were no differences between groups in the ratio of capillaries to muscle fibers. (**E**) The capillary supply area was greater in MCT and MCT+BB than CON, but was reduced in MCT+BB compared to MCT. *n* = 6 hearts per group. * *p* < 0.05, ** *p* < 0.01, *** *p* < 0.001.

Histology was conducted on cryosections of RV myocardium from CON, MCT, and MCT+BB rats. 10 μm thick sections were stained with fluorescein-conjugated lectin to label the capillary network and muscle fiber boundaries (Figure 1A). Capillary locations were digitized, and the capillary supply area, in which all points were closer to a central capillary than to any other capillary, was computed using Voronoi tessellation (Figure 1B). Capillary density was reduced by 45% in MCT and by 33% in MCT + BB compared to CON (*p* < 0.001), but was increased in MCT + BB compared to MCT (*p* < 0.05) (Figure 1C). This could indicate capillary rarefaction or be a consequence of angiogenesis failing to match RV myocyte hypertrophy [37]. The ratio of capillaries to muscle fibers was not different between groups (Figure 1D). The mean capillary supply area was increased by 81 % in MCT (*p* < 0.001) and by 53 % in MCT+BB (*p* < 0.01) compared to CON, but was reduced by 16% in MCT+BB compared to MCT (*p* < 0.05) (Figure 1E). This indicates that angiogenesis did not match muscle fiber hypertrophy in MCT, but that BB ameliorated the increase in the capillary supply area. 

**Figure 2:**
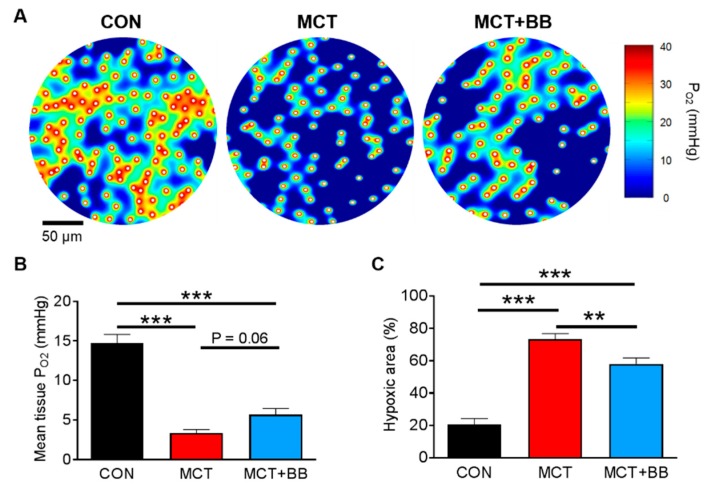
Computer modelling predicts RV hypoxia in pulmonary arterial hypertension (PAH) is ameliorated by beta-1 blocker (BB) treatment. (**A**) Example heat maps showing areas of high P_O2_ surrounding capillary locations (white circles) and hypoxic regions distal to the capillaries. (**B**) Mean tissue P_O2_ was reduced in MCT and MCT+BB compared to CON. (**C**) The hypoxic area was greater in MCT than CON, but was reduced in MCT+BB compared to MCT. ** *p* < 0.01, *** *p* < 0.001. *n* = 6 rats per group.

To investigate whether perfusion mismatch could lead to tissue hypoxia, a finite element model of O_2_ diffusion and consumption in the myocardium was fitted to the experimentally determined capillary locations [39,40]. The model was implemented in Matlab, and is available with a user-friendly graphical user interface [41]. Figure 2A shows exemplary heatmaps of tissue P_O2_ surrounding the capillaries (indicated by white circles). It is apparent that a greater proportion of MCT tissue is predicted to be at low P_O2_ compared to CON tissue. The mean tissue P_O2_ in MCT was 73% less and in MCT + BB was 61% less than that found in CON (*p* < 0.05) (Figure 2B). Mean tissue P_O2_ was not significantly different in MCT compared to MCT+BB (*p* = 0.06). When tissue P_O2_ is greater than ~0.5 mmHg, the tissue O_2_ flux is approximately constant, whereas below this critical level O_2_ utilization falls sharply [42]. Using this criterion for hypoxia, the percentage of hypoxic tissue was significantly increased in MCT compared to CON (*p* < 0.05). The hypoxic area was also greater in MCT+BB compared to CON; however, the area was reduced compared to MCT alone (*p* < 0.05) (Figure 2C).

We investigated whether differences in O_2_ extraction could influence the model results. Proliferation of interstitial fibrosis could act as a barrier to O_2_ diffusion from the capillaries to muscle fibers [43,44], however we found no difference between groups in the extent of fibrosis assessed by picrosirius red staining (CON: 3.2 ± 0.4%; MCT: 2.5 ± 0.3%; MCT+BB: 2.7 ± 0.4%; *p* = 0.44). A reduction in mitochondrial mass could reduce tissue O_2_ utilization and ameliorate hypoxia [45]. Citrate synthase activity (a marker of mitochondrial mass [46]) was measured in RV homogenates taken from the same hearts used for histology. Citrate synthase activity did not differ between groups (CON: 5.39 ± 0.45 IU/mg; MCT: 4.37 ± 0.47 IU/mg; MCT + BB: 4.30 ± 0.42 IU/mg; *p* = 0.19), indicating no change in overall mitochondrial density. These data indicate that tissue hypoxia, which is ameliorated by chronic BB treatment, may be a limiting factor in the RV of MCT rats.

**Figure 3:**
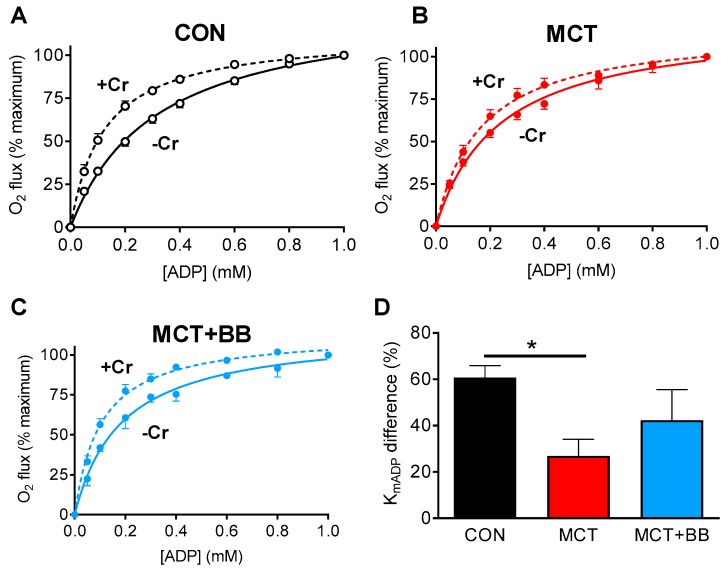
Coupling of mitochondrial respiration to the creatine kinase (CK) system is decreased in MCT. (**A**–**C**) The increase in O_2_ consumption of permeabilized RV myocytes upon sequential titration of ADP was measured in the absence (solid line) or presence (dashed line) of Cr. (**D**) The relative difference in K_mADP_ with Cr compared to without Cr was greater in CON than MCT, but not greater than in MCT+BB. * *p* < 0.05. *n* = 8 CON, 5 MCT, 3 MCT + BB independent replicates.

The CK system is important for distributing energy generated by mitochondria to the myofilaments, especially during hypoxia [25]. We previously reported decreased expression of CK-mt in MCT rats [29], and wanted to investigate whether this causes a functional impairment in respiration that could affect energy availability in hypoxic regions. Mitochondrial respiration was measured in permeabilized isolated RV cardiomyocytes in response to increasing concentrations of ADP [27,47]. The apparent K_mADP_ in the absence of Cr was not different between groups (*p* > 0.05). When the experiment was repeated in the presence of 25 mM Cr (to activate CK-mt), there was a shift towards a lower K_mADP_ in CON cells (–Cr: 0.35 ± 0.05 mM; +Cr: 0.14 ± 0.02 mM; *p* < 0.05), but no difference in MCT cells (–Cr: 0.25 ± 0.03 mM; +Cr: 0.18 ± 0.03 mM) or MCT+BB cells (–Cr: 0.20 ± 0.04 mM; +Cr: 0.11 ± 0.02 mM) (Figure 3A–C). The relative difference in K_mADP_ with Cr in CON cells (61% reduction) was greater than in MCT cells (27% reduction) (*p* < 0.05) (Figure 3D). This indicates reduced functionality of the CK system to activate respiration in MCT cells. The difference in K_mADP_ in MCT+BB in the presence of Cr (42%) was not different to CON or MCT (*p* > 0.05), suggesting CK-mt function in MCT+BB is intermediate between groups. 

## 3. Discussion

We demonstrate that a reduction in capillary density is predicted to result in severe myocardial hypoxia in the failing RV in PAH. Chronic BB administration reduces RV hypertrophy in PAH [34,37], and we now show that it also ameliorated the extent of hypoxia in BB-treated rats. We also show CK-mt function is impaired in MCT RV cells. This may alter the balance of high-energy phosphates within the cell and reduce the rate of energy turnover at ATPases. The changes seen are likely to reduce RV contractile performance in PAH, particularly during increased demand [29]. These data suggest that a beta-adrenoceptor blockade is a viable strategy to sustain the contractility of the RV in PAH, by improving the metabolic state of the myocardium.

**Figure 4:**
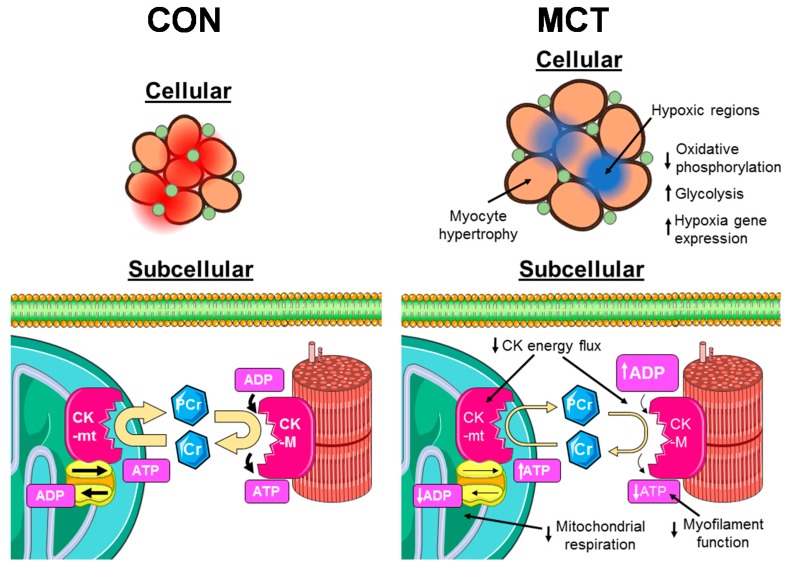
Cartoon representation of proposed changes in structure and function of RV myocytes in PAH. In CON cells (**left**), the densely packed capillary network delivers O_2_ evenly throughout the myocardium, maintaining a high P_O2_ (red regions). Mitochondria utilize O_2_ for oxidative phosphorylation, generating ATP. Mitochondrial-bound CK (CK-mt) use ATP to generate PCr, which diffuses to cytosolic sites of high ATP turnover, such as myofilaments, where cytosolic CK converts it back to ATP. This maintains a high ATP:ADP ratio in the myofilaments, allowing them to work properly, and a low ATP:ADP ratio in mitochondria, promoting ATP synthesis. In MCT cells (**right**), RV myocyte hypertrophy increases the diffusion distance of O_2_ from the capillaries, resulting in hypoxic cores developing within muscle fibres (blue regions). These areas are unable to meet metabolic needs entirely aerobically, so they become reliant on glycolysis. Energy transport to hypoxic cores is further impaired by reduced expression of CK isoforms, resulting in accumulation of ADP in myofilaments and reduced mitochondrial respiration. Cartoon was created using Servier Medical Art by Servier, which is licensed under a Creative Commons Attribution 3.0 Unported License (http://www.servier.com/slidekit)

The schema presented in Figure 4 highlights the changes we observed in PAH and some functional implications. Increased maximum diffusion distance between capillaries and muscle fibers resulted in a proliferation of hypoxic regions. This would impair mitochondrial ATP generation in these regions and could be driving increased expression of hypoxia-related genes and increased glycolysis [17,18]. At the subcellular level, reduced activity of CK isoforms decreases high energy phosphate transfer between mitochondria and myofibrils in the hypoxic regions. This reduces the rate of ATP turnover at sites such as the myofibrils and SERCA, thus impairing contraction and preventing them contributing to cardiac work.

### 3.1. Insufficient Capillary Supply Produces Extensive Myocardial Hypoxia in Monocrotaline Rats

Hypertrophy of the RV was apparent in MCT rats, but was reduced following BB treatment (Table 1). The increase in RV myocyte hypertrophy without an increase in the capillary:fiber ratio increased the mean area of myocardium supplied by each capillary, leading to hypoxia. Hypoxia triggers changes in the transcription factors regulating angiogenesis, which follow a complex expression pattern possibly related to disease severity and temporal state [12,34,48]. Decreased proangiogenic micro-RNAs and increased expression of inhibitors of VEGF and angiopoetin-1 have also been reported to increase in latter stage heart failure, which could explain the lack of angiogenesis [13,21,22].

A steep O_2_ gradient between the cell periphery and core of cardiac myocytes is expected to exist, due to densely packed subsarcolemmal mitochondria [49]. This gradient should be more apparent in a hypertrophic myocardium with greater O_2_ diffusion distances. By applying a computer model of O_2_ flux in the myocardium to our experimental findings in heart failure, we were able to quantify the effect of capillary density reduction on myocardial hypoxia. The three-fold increase in the hypoxic area in MCT rats implies an increase in tissue fraction, which is unable to contribute substantially to ventricular work. These areas will not necessarily become necrotic, as non-contracting myocytes with low O_2_ demand could be sustained through anaerobic glycolytic pathways [25]. BB reduced the proportion of hypoxic tissue, mainly by limiting myocyte hypertrophy (Figure 1E and Table 1). BB may have further benefits in vivo by reducing heart rate, and therefore O_2_ demand, and increasing diastolic coronary perfusion [5,19].

Coronary artery disease is increasingly prevalent in PAH patients [50], and occurs around four times more frequently among that group than in the general population [51,52]. Coronary artery remodeling also occurs in parallel with the development of MCT-induced PAH, and is associated with reduced coronary perfusion [53]. Modulation of transcription factor activity by scaffold proteins, e.g., bromodomain protein 4 (BRD4), is implicated in coronary artery disease by altering the balance of proliferation and apoptosis of smooth muscle cells [53]. Decreased RV coronary perfusion is likely to exacerbate the microcirculatory impairment we identify. BB may protect against the ischemic consequences of coronary artery disease in the RV, as in the LV [54].

Our observations suggest that normalizing the capillary supply area in PAH would be beneficial. One way to achieve this would be augmenting angiogenesis. Exercise improves cardiac function [55], and increases angiogenesis and oxygen uptake efficiency [56]. Exercise therapy now has cautious recommendation in PAH guidelines [9]. We have shown that exercise has a beneficial effect in the MCT rat model [57].

### 3.2. Reduced Mitochondrial Synthesis of Phosphocreatine in Monocrotaline Rats

The CK system has a vital role maintaining a high [ADP] in mitochondria and a low [ADP] at the myofibrils. The less negatively charged and smaller molecular weight phosphocreatine (PCr) has greater cytosolic mobility than adenine nucleotides [58,59], so it acts as a shuttle of high energy phosphates to sites of high ATP turnover, such as the myofilaments. This also allows sudden changes in cellular energy demand to feed back to mitochondria, which sense an increase in cellular [ADP] and increase ATP production accordingly, e.g., during periods of increased heart rate. We identified uncoupling of the CK system with mitochondrial respiration, which was likely due to decreased expression of CK-mt [29]. This may limit the ability of mitochondria to respond rapidly to a sudden increase in work, as was demonstrated in MCT RV trabeculae [60]. A small decrease in Cr and PCr levels, which could further decrease CK flux in vivo, has been reported in the MCT model as well as other animal heart failure models and human heart failure [61,62,63]. The mechanical efficiency of the failing RV is also reduced, which will further increase the magnitude of the energy demand mismatch [64,65].

### 3.3. Limitations

Different classes of BB may have distinct effects in PAH. Carvedilol has mixed alpha and beta-1/2 adrenoceptor blocking ability, and nebivolol has pulmonary vasodilatory ability. Both were found to reduce afterload and adverse remodeling in experimental PAH [34,36]. Metoprolol is a beta-1 selective blocker, and is therefore predicted to be more specific to the myocardium rather than vasculature. We chose metoprolol to investigate the direct actions of a beta-blockade on the myocardium without confounding effects due to afterload reduction [35]. The response to BB may also depend on the etiology of PAH. BB are currently not recommended in Group 1 pulmonary hypertension (PAH), whereas BB are more likely to be prescribed in Group 2 pulmonary hypertension (secondary to LV failure), due to co-morbidities. MCT is known to affect the lungs, liver, and kidneys [66]. Increased wet lung weight is indicative of pulmonary edema; however, proliferation of lung tissue will also increase the dry lung weight [67].

### 3.4. Conclusion

We demonstrate that BB treatment reduced hypertrophy in experimental PAH, resulting in a reduction in hypoxic myocardium. This was accompanied by a partial improvement in functional coupling between mitochondria and the CK energy system. These improvements are likely to act in concert to favorably affect energy availability in RV myocytes, which will help maintain cardiac function for longer. These data add to previous findings of improvements in excitation–contraction coupling, electrical remodeling, and inflammation following BB in experimental PAH [34,35,37]. BB were well tolerated in Group 1 PAH patients under close clinical supervision, and showed preserved cardiac function and reduced RV glycolysis after six months of treatment [33]. Together with our preclinical findings, this supports the notion that BBs may be beneficial in PAH by reducing adverse cardiac remodeling, although further testing in both preclinical and carefully controlled clinical settings will be required to confirm this. 

## 4. Materials and Methods

All experiments were conducted in accordance with the Animals (Scientific Procedures) Act (1986), the European Parliament Directive 2010/63/EU, and with local ethical approval (70/8399, 14 February 2015).

We used the well-characterized monocrotaline (MCT) rat model of PAH, described in detail in [37]. Briefly, a single intraperitoneal injection of MCT (60 mg/kg) into male Wistar rats (200 ± 20 g) causes a progressive increase in pulmonary vascular resistance and RV systolic pressure, eventually leading to RV failure. The beta-1 adrenergic receptor blocker metoprolol (10 mg/kg) was administered daily by voluntary syringe feeding, starting on day 15 post-MCT injection, as described previously [37,38]. MCT animals were killed upon showing signs of heart failure, such as weight loss on consecutive days, dyspnea, and piloerection. These symptoms have been validated as indicative of RV failure in this model [68,69,70,71,72]. 

### 4.1. Histology

Tissue was prepared for histology, as described in [37]. Tissue sections 10 μm thick were plated on microscope slides and fixed in acetone for 5 min on ice. Slides were incubated for 1 h with fluorescein-conjugated lectin from *Griffonia simplicifolia* (Vector Laboratories, Burlingame, CA, USA), at a final concentration of 10 µg/mL in phosphate-buffered saline. Slides were imaged on a Nikon Eclipse E600 microscope with a Nikon Plan Fluor 20X objective, using 487 ± 10 nm excitation light and fluorescence collected at 533 ± 20 nm. Capillaries were identified by the bright focal staining pattern and their location digitized. Muscle fiber boundaries were identified by the less intense staining of the glycocalyx surrounding the cell membrane. Fibrosis was assessed using picrosirius red staining, as described previously [29]. Results from two to five sections per heart were averaged to calculate the representative value for each animal, which was used for subsequent statistical analysis.

### 4.2. Computer Modelling O_2_ Distribution

We used a finite element model of O_2_ diffusion implemented in Matlab and described in detail in [39,40,41]. Model parameters used were in accordance with published literature [73], and were kept constant for all groups.

### 4.3. Mitochondrial Respiration in Isolated Myocytes

Single RV myocytes were isolated by enzymatic digestion of the heart on a Langendorff apparatus, as previously described [74]. Cells were resuspended in a respiration recording solution containing the following (in mM): 110 sucrose, 60 K-lactobionate, 20 HEPES, 20 taurine, 10 glutamate, 10 KH_2_PO_4_, 3 MgCl_2_, 0.5 malate, 0.5 EGTA, 1 mg/mL bovine serum albumin (pH 7.1 with KOH). Equal volumes of cell suspension were added to both chambers of an Oxygraph-2k (Oroboros Instruments, Inssbruck, Austria). In one chamber, 25 mM creatine was added to the media. The sarcolemma was selectively permeabilized with 50 μg/mL saponin injected directly into the chamber using a Hamilton syringe. O_2_ flux was recorded in response to sequential titrations of ADP [27,47]. Temperature was maintained at 37 °C, and O_2_ concentration was maintained between 200 and 400 μM throughout the experiment. Data were normalized to O_2_ flux in the absence of ADP (0%) and at the maximal flux in 1 mM ADP (100%). The apparent affinity for ADP (K_mADP_) was determined by fitting Michaelis–Menten kinetics to the normalized data.

### 4.4. Enzyme Activity Assays

RV samples were homogenized on ice in a buffer containing, in mM, 5 HEPES, 1 EDTA, 5 MgCl_2_, and 0.1% Triton-X100 [75]. Protease inhibitors (cOmplete Protease Inhibitor Cocktail, Roche, Bazel, Switzerland) and phosphatase inhibitors (Halt Phosphatase Inhibitor Cocktail, Thermo Scientific, Waltham, MA, USA) were added to the buffer immediately before use. CK activity in samples was measured spectrophotometrically from the production of NADPH, according to the Rosalki assay [76], with absorption measured at 340 nm. Next, β-mercaptoethanol (1 mM) was added prior to the CK activity assay, to prevent the oxidation of CK. Citrate synthase activity was measured using a spectrophotometer (Varioscan Flash, Thermo Scientific, Waltham, MA, USA) according to the enzyme assay described by Srere [77]. Enzyme activity was expressed in international units per mg protein (IU/mg).

### 4.5. Statistics

Data are presented as mean ± standard error of mean (SEM). Parametric ANOVA was performed on normally distributed data—otherwise, non-parametric equivalents were used. Holm–Sidak post hoc tests used were for parametric ANOVA, or Dunn’s (unequal group sizes) for non-parametric ANOVA. The number of independent replicates (hearts or cell isolations) are given in figure legends, and *p* < 0.05 was considered statistically significant.

## Figures and Tables

**Table 1:** Body and organ characteristics of control (CON), monocrotaline (MCT), and beta-1-selective blocker metoprolol (MCT + BB) groups.

	CON	MCT	MCT+BB
Body weight (g)	330 ± 7	294 ± 13	273 ± 10 **
HW:BW (mg/g)	3.90 ± 0.13	6.22 ± 0.34 ***	5.07 ± 0.34 *^,†^
RV:BW (mg/g)	0.75 ± 0.05	1.42 ± 0.12 ***	0.90 ± 0.07 ^††^
LV + S:BW (mg/g)	2.78 ± 0.17	3.00 ± 0.19	2.96 ± 0.09
Lung:BW (mg/g)	7.53 ± 0.82	10.3 ± 0.3 *	10.2 ± 0.6 *
Liver:BW (mg/g)	39.6 ± 1.3	40.6 ± 2.8	49.2 ± 1.8 *^,†^

HW: heart weight; BW: body weight; RV: right ventricle, LV + S: left ventricle + septum. * *p* < 0.05, ** *p* < 0.01, *** *p* < 0.001 vs. CON, ^†^ *p* < 0.05, ^††^ *p* < 0.01 vs. MCT. *n* = 7 CON, 5 MCT, 5 MCT + BB rats per group.

## Abbreviations

BB	beta-adrenergic receptor blocker
CK	creatine kinase
CON	saline-injected control
Cr	creatine
MCT	monocrotaline
MCT+BB	monocrotaline + beta blocker
PCr	phosphocreatine
SERCA	sarco(endo)plasmic reticulum Ca^2+^ ATPase
